# First Annual Report Bureau of Animal Industry

**Published:** 1885-07

**Authors:** 


### REVIEWS.

THE FIRST ANNUAL REPORT BUREAU OF ANIMAL INDUSTRY.

We have before us the First Annual Report of this newly-created bureau, with reference to the contagious diseases of the domestic animals in the United States for the year 1884. It is a work which should be in the hands of every stock raiser in the country, and especially every veterinarian. We recommend those of our readers that have not yet received this book to at once write the Agricultural Department, at Washington, for a copy; without it they cannot know what has been done, nor what needs to be done, nor of the many difficulties which still lie in the way of doing good work. Every unbiased mind must become convinced of several things in reading this report:

1st.—That we, as a profession, have a most creditable representative in Dr. D. E. Salmon, Chief of the Bureau;

2d.—That it is the best and most complete report yet issued by our government upon this important subject;

3d.—That a much more extensive veterinary police organization over the country is necessary than we have at present, and that we must endeavor to make not only the United States Government, but the respective State governments, realize the necessity of giving employment to as many qualified men as possible if they would really do their duty to the citizens of this country.

The report, in every way, confirms the views we have been advocating for the past ten years; so much so, in fact, that many of our words would seem to have borne the spirit of true prophecy.

In this JOURNAL, but especially in our book "On the Relation of Animal Diseases to the Public Health," we have repeatedly drawn attention to the futility of State work so long as there was no concentrated action, or the doctrine of State rights was adhered to, and of the absolute necessity of one and the same code of laws for every State in the Union, and one veterinary police force emanating from a head-centre at Washington. We find that experience has more than endorsed our teachings.

Our ideas of the importance and urgent necessity of a national veterinary police also find their confirmation in Dr. Salmon's experiences, which he sums up as follows:

"A national direction of the work for the extermination of pleuro-pneumonia," (which he estimates costs the country in direct and commercial injury about $4,000,000) "would overcome at once the discouraging features which have done so much to prevent the efforts of the individual States from being effective. With inspectors in every infected State, the shipment of diseased cattle would soon cease, new outbreaks would thus be prevented, and the danger which has so long menaced the great cattle interests of the country, would be removed. The work would be more thorough, because those engaged in it would not be either directly or indirctly dependent upon the good-will of the interested cattle owners for their positions, and the plea of inability to pay for the diseased cattle which should be slaughtered would also be overcome. These have been the principle obstacles to the success of State action, and practically they are so great as to make it next to impossible for the States alone to free themselves from this plague."

At last we have a veterinarian at Washington who is not afraid to publish the exact condition of things in his report.

The year 1884 was marked by a great and dangerous extension of that bovine pest, contagious pleuro-pneumonia, over the country. Up to this time it had been limited to a few of our Atlantic sea-board States. It also extended west over the Alleghenies, for the first time being taken from Maryland with some grade Jerseys, to Troy, Ohio, from whence it was transported to Virginia, Ill., and by means of an extensive sale of cattle, dispersed largely over Illinois, and found its way into Kentucky. Some of these cattle from Virginia, Ill., also went into Missouri, and as we have lately heard of the disease appearing there, and as we know well its peculiarly insidious nature, and that it frequently occurs in so mild a form in individual cases as scarcely to attract attention, is it not possible, and also probable, that this whole extension is one chain extending originally from Maryland, with a link missing here and there, to Missouri at present, and no one knows where it will next show itself.

The question that at once comes to mind is: Who is to blame for all this damage? It cannot be the United States Government, for the report shows that the veterinary authorities have done all they could under existing circumstances. The blame is to be found in the want of intelligence of the people of the country as we find it represented by their respective legislators.

The State authorities have not had the courage to admit the truth, because of their fear of losing votes or influence, or injuring the animal industry of their State. They should follow the brave example of the United States authorities, for in this report we find that the truth will come out and always must, eventually. The report calls especial attention to the fact that there is a steadily increasing trade in calves and grade cattle from the infected States to the west, which must certainly be a constant source of danger that can only be checked by the organization of a trustworthy veterinary police in the afflicted States Even now the governors of some of the Territories and western grazing States are threatening to quarantine cattle from eastern States, unless accompanied by certificates from State veterinarians. It would be interesting to know how many of the eastern States have trustworthy and competent State veterinarians.

Although no veterinarian of any education would utter a doubt as to the contagiousness of this bovine lung pest, still it has been found that history repeats itself in this country in this as with many other things, and that many reports were being circulated with regard to the non-contagiousness of this disease. It was therefore right that the Bureau should take cognizance of this fact and show by direct experiment that the bovine lung pest is now, as it always has been since known to history, a strictly contagious disease.

The malignant character of the inficiens when introduced into the subcutaneous tissues was well shown by injecting small quantities of the same into the tissues in the vicinity of the shoulder. Intense inflammatory reaction resulted, leading to the death of two out of three animals.

It was also found that animals taken from a region in which the disease was known to have existed were not so susceptible to infection from cohabitation with known diseased ones as those from entirely uninfected districts. Whether or not the first had had the disease in the mild form it often assumes was not to be ascertained. Of four healthy cows from West Virginia exposed to contagion in the above manner from May 1 to 5, No. 1 had a severe cough, with pulmonary complication, and a temperature of 105 1-5 on the 23d of May. No. 2, the same on the 1st of June; No. 3, on May 25th; No. 4, on June 9th. All of them exhibited the known lesion of contagious pleuro-pneumonia at the microscopical examination.

At Barren Island, a place where there had never been any disease, the government built a stable and introduced 18 cows and 13 calves from Canada, where the disease has not yet appeared. With these cattle were placed five diseased ones from that pest-hole, Brooklyn.

Of these 31 animals, 21 contracted the disease between the 20th of Sept., 1884, and Jan. 3, 1885. European experience has shown that 21.5 per cent. of the animals exposed to contagion acquired this disease, and in this country, so far as can be estimated, the percentage of actual infection is approximately the same—28.2 per cent.

In this connection, we would mention that Dr. J. F. Winchester, veterinary surgeon at Lawrence, Mass., and lecturer at the Massachusetts State Agricultural School, recently sent to us a small piece of lung from a cow that had the strongest resemblance to contagious pleuro-pneumonia that we have ever seen. Our diagnosis was primarily broncho-pneumonia, extending to interstial, and finally becoming parenchymatos also. Pleuritis was also present; in the letter that came after the specimen had been examined, he does not state anything as to an effusion having been present in the pleural cavities, but that the disease "had been caused through drenching the cow." The marbled appearance was quite marked, but on making a section of diseased parts it was not followed by the flow of serum that occurs in recent pleuro-pneumonia. The parenchymatos affection was more evenly distributed than in the contagious form, and there was no indications of approaching necrosis, as in that disease, but, most important of all, there was none of that *œdematous anœmic atalutasis*, so frequent in the contagious variety, which is to be looked upon as the cause of the necrosis of parts of the lungs and breaking down of the tissues.

To us one of the most interesting communications in this report is the description of the experiment station and laboratory of the Bureau, situated in the vicinity of Washington, Dr. Rose being the superintendent of the former, as Dr. Salmon must be of the latter. As known to our readers, we have long been advocating the necessity of such an institution.

The corner-stone having been laid, there is nothing in the way of Dr. Salmon making his name immortal by persistently using his position and abilities to influence Congress to the foundation of a National School of Comparative Medicine at Washington, as we stated in an editorial in our last issue.

An article for which we have waited with the greatest interest is Dr. Salmon's contribution upon the outbreak in Kansas in the winters of 1883-4, which has been designated under so many names or causes, but which was either foot and mouth disease, or as Dr. Salmon calls it, ergotism. We must confess that while reading Dr. Salmon's article on the subject that the views and descriptions given by him have served materially to confirm our previous opinion that it could not have been the contagious foot and mouth disease. On the other hand, we have been favored with the unpublished report of Mr. McEachran, of Montreal, the head of the veterinary police of Canada, who as positively asserts that the disease was foot and mouth.

We must confess that we still adhere to Dr. Salmon's conclusions, notwithstanding our great respect for the abilities of our friend in Canada. We have heard that Dr. Holcomb has also made a report to the government of Kansas in which he adheres to his first expressed opinion that the disease was foot and mouth. This is certainly a case where the "doctors" that were present and in person examined all the conditions and collateral circumstances did not agree. We have, therefore, decided not to consider this part of the report of the Bureau at present, but to obtain Dr. Holcomb's report and give our readers a synopsis of the evidence of all these professionals, leaving them to judge for themselves; one thing they may be sure of, the careful study of this part of Dr. Salmon's report is the best article on ergotism that they can gain access to, for his position at Washington gives him access to the National Medical Library, the finest in the world, an advantage which none of us possess, and he has used it with great benefit to all of us.

This volume also contains several reports from various others of its agents with reference to the animal interests of the country such as stock-raising on the plains, transportation, slaughtering, etc.

Mr. James Law gives a condensed report of the occurrences at the late Veterinary Congress at Brussels, in which he advocates the ideas of a veterinary police and education of veterinarians.

There is also an interesting translation, accompanied with illustrations, of syngamus trachealis, of an article by Meguin, a noted French helminthologists, upon the gapes in fowls.

There is also a report on trichiniasis which, while instructive, adds nothing to our knowledge, the government having still overlooked this important question, not having inaugurated any investigations into the origin of trichinæ outside the porcine organism; a duty which it is to be hoped the present Commissioner will fulfill.

The report is well printed and illustrated, and should be read by every veterinarian in the country, for upon the subjects on which it treats it is a better text-book than any other they have access to, and contains much interesting and collateral information. B.

